# A case of ascetic fluid *Mycobacterium aubagnense* infection in a patient with severe peritoneal effusion

**DOI:** 10.1186/s12879-023-08041-1

**Published:** 2023-02-07

**Authors:** Yuanqin Du, Jingjing Huang, Yaobin Nong, Ruixi Zhong, Ronghuo Zhu, Wenxuan Song, Hongna Huang, Qinwen Tan, Jian Xu, Xiyu Xiao, Juhong Jia

**Affiliations:** 1 Guangxi University of Chinese Medicine, Nanning, China; 2 Department of Spleen and Stomach Liver Diseases, The First Affiliated Hospital of Guangxi University of Traditional Chinese Medicine, Xianhu District, Nanning, China; 3 Guangxi Key Laboratory of Translational Medicine of Integrated Traditional Chinese and Western Medicine, Nanning, China; 4 Guangxi Key Laboratory of Molecular Biology of Preventive Medicine of Traditional Chinese Medicine, Nanning, China

**Keywords:** *Mycobacterium aubagnense*, Non-tuberculosis mycobacteria, Ascites

## Abstract

**Background:**

*Mycobacterium aubagnense,* which was first characterized in 2006, is a non-tuberculosis mycobacterium (NTM) that has only been isolated from respiratory secretions and joint fluid. With only four cases globally, the microbe has rarely been reported in human clinical cases and the strain has not been isolated from ascites.

**Case presentation:**

To the best of our knowledge, this is the first time that *M. aubagnense* has been isolated from ascites samples of a patient with severe peritoneal effusion and normal liver functions. Anti-NTM therapy with moxifloxacin, ethambutol, and isoniazid combined with furosemide and spironolactone diuretic therapy relieved the symptoms after six months.

**Conclusions:**

Increased puncture and drainage of ascites combined with diuretic treatment did not significantly relieve the ascites, leading to relapse with aggravated symptoms. The subsequent anti-NTM treatment with moxifloxacin, ethambutol, and isoniazid alleviated the degree of ascites. Therefore, we postulated that *M. aubagnense* infection was the potential cause of the difficult reduction of ascites in this patient. However, the ascites repeatedly occurred in the patient, which was attributed to *M. aubagnense* resistance due to insufficient medication time and repeated medication. The patient's underlying diseases may also result in ascites. Therefore, there is a need for careful analysis of the clinical significance of *M. aubagnense*.

## Background

*Mycobacterium aubagnense*, a non-tuberculosis mycobacterium that is isolated from respiratory secretions and joint fluid, was first reported in 2006 [1]. It is commonly referred to as Aubagne, after the city where the first patient was identified. It is closely related to *Mycobacterium mucogenicum,* with a difference of 11 bases (99.2% similarity). Gene sequences of the two strains are also closely related to those of *Mycobacterium phocaicum,* with differences of less than 5 to 15 bases; hence, they are classified into the *Mycobacterium mucogenicum* group ^[[[2]]]^.

## Case presentation

The 61 year old patient is a retired worker who has long lived in the city far away from natural environments such as rivers and wetlands. The patient had no smoking or drinking habits and had not undergone surgery. Due to abdominal fullness and recurrent lower limb edema, he was treated several times in the local hospital in 2019–2021. In April 2021, he was hospitalized at a local hospital. Abdominal computed tomography revealed ascites, and he was diagnosed with “viral Hepatitis B, cirrhosis, and ascites”. After the administration of diuretics to discharge ascites through puncture, there was no significant improvement in the patient’s condition. In May 2021, the symptoms aggravated, leading to his transfer to our hospital in July 2021. Physical examination revealed abdominal distention, positive fluid wave tremors, positive migratory dullness, no tenderness, rebound pain in the whole abdomen, and moderate pitting edema in both lower limbs. A blood sample was taken for analysis. The albumin level was 41.3 g/L, with normal liver functions. Renal function analysis revealed that urea, creatinine, and uric acid levels were 17.80 mmol/L, 149 μmol/L, and 64 μmol/L, respectively. The Hepatitis B virus-DNA test and the human immunodeficiency virus (HIV) test were negative. B-type ultrasonic inspection of ascites showed ascites (maximum anterior–posterior diameter of 7.5 cm), while epigastric CT scan revealed cirrhosis with ascites. Electronic gastroscopy further revealed portal hypertensive gastropathy. The extracted ascites were pale yellow and cloudy in texture, with 3.05 × 10^8^/L white blood cell counts and 27.1 g/L albumin levels. The mucin of ascites serous membrane was characterized as weak positive. His liver functions were normal, portal vein pressure was slightly high, and the ascites were turbid with a high white blood cell count. The ascites were considered to have been caused by spontaneous peritonitis. However, cultures of the subsequent ascites were negative for bacteria and fungi, specific T-cell immune responses to *Mycobacterium tuberculosis* were positive, and the patient had no rebound tenderness. The patient was also considered to have tuberculous peritonitis; however, X-ray analysis did not show any signs of tuberculosis, with ascites adenosine deaminase levels determined to be 4.1 U/L. Eventually, the ascites were considered to have been caused by water fluid retention due to renal failure. Therefore, the patient was subjected to three times ascites drainage (draining 8000 mL of fluid), after which he was administered with furosemide and spironolone for diuretic drainage and discharged from the hospital. He was also prescribed diuretics, which he continued taking after discharge.

In October 2021, the patient was hospitalized again due to ascites and abdominal distention. B ultrasonography of ascites revealed ascites with a maximum anterior diameter of 9 cm. Blood sample analysis revealed normal liver functions. However, white blood cell counts in ascites were 4.09 × 10^8^/L, and ascites mucin was positive. In addition, the *Mycobacterium tuberculosis*-specific t-cell immune responses were positive, the erythrocyte sedimentation rate was 20 mm/h, ascites adenosine deaminase levels were 5U/L, and the ascites cultures were negative for bacteria and fungi. Chest CT scan was negative for tuberculosis infection. The patient was advised to undergo tuberculous/non-tuberculosis mycobacteria identification, which he rejected. Therefore, tuberculous peritonitis was temporarily considered, and anti-tuberculosis medications, including moxifloxacin, ethambutol, and isoniazid administered. During hospitalization, the ascites were repeatedly extracted. After discharge, the anti-tuberculous drugs were continued for 6 months, and furosemide and spironolacone diuresis were given for more ascites. However, the patient often stopped taking the drugs due to nausea and vomiting.

**Fig. 1:**
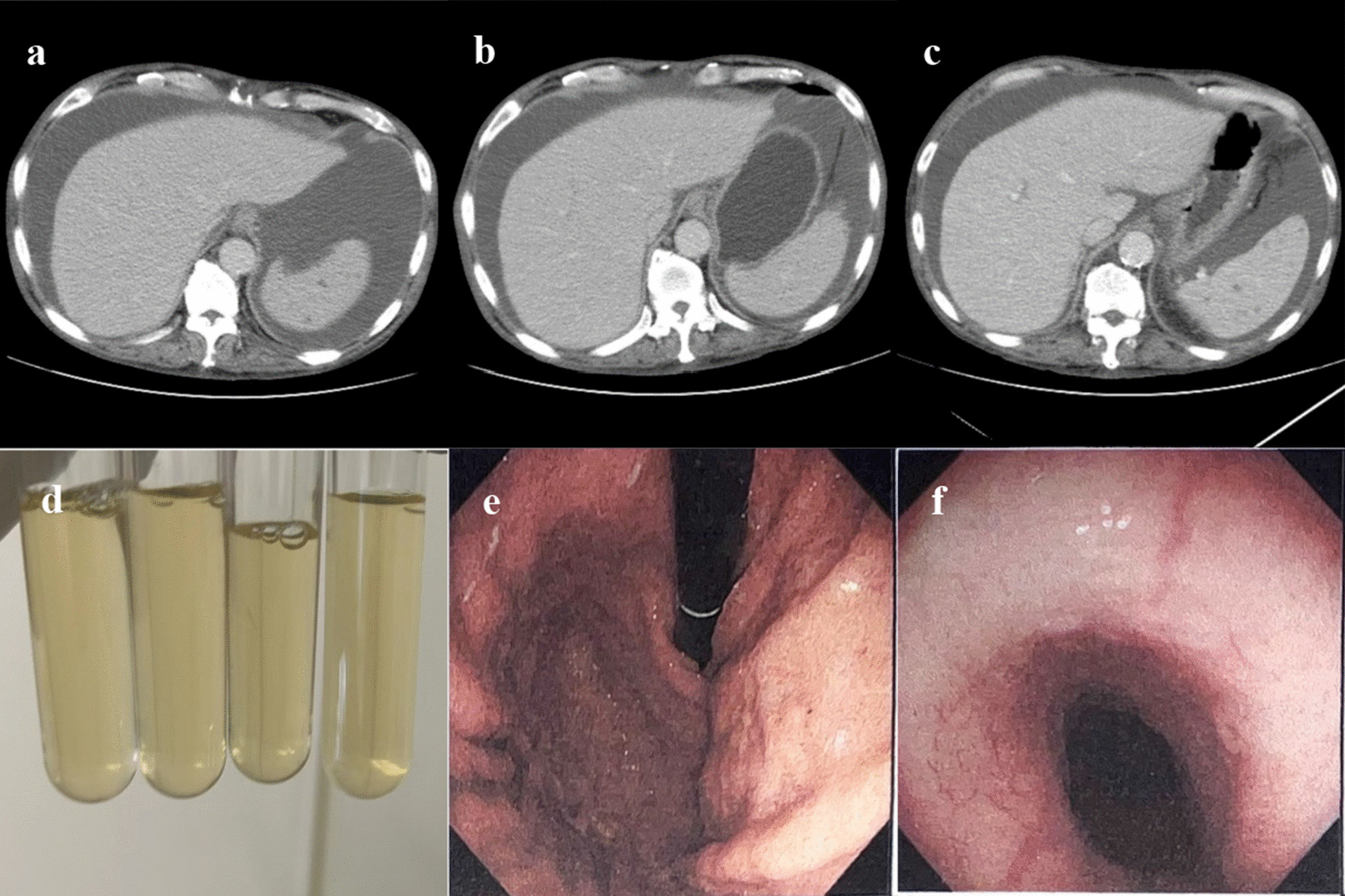
**a** A CT scan micrograph showing massive ascites on admission; **b** A CT scan micrograph showing persistent ascites; **c** A CT scan micrograph showing reduced ascites after anti-NTM treatment; **d** Ascites from the first peritoneal punch; **e** Gastric fundus with no obvious varicose veins under digestive endoscopy; **f** The esophagus with no obvious varicose veins under digestive endoscopy

In May 2022, the patient was hospitalized for the third time. He had a flat abdomen, and an upper abdominal CT scan revealed that the degree of ascites was significantly reduced. The anti-nuclear antibody and tuberculosis antibody tests were negative, but *Mycobacterium tuberculosis*-specific T-cell immune responses were positive. A negative test result for antinuclear antibodies ruled out autoimmune diseases. The accuracy of the tuberculosis antibody detection assay, which is often used to assist in diagnosis, was insufficient. A negative result does not rule out *Mycobacterium tuberculosis* (MTB) infections. The positive MTB-specific T-cell immune responses indicate past or recent MTB infections. The ascites samples were extracted and sent to Zhejiang Shengting Biotechnology Limited Company to identify MTB and the 168 NTM species using the high-throughput nanopore system. Full length sequencing was performed to identify the 16 anti-MTB drug resistance genes, including isoniazid, quinolones and ethambutol, and 5 anti-NTM drug resistance genes, including clarithromycin and Amica magnitude. The *M. aubagnense* was detected, but no drug-resistance genes were detected. Therefore, given the difficulty associated with the elimination of *Mycobacterium obaneum-*associated ascites, administrations of moxifloxacin, ethambutol, and isoniazid anti-NTM treatments were continued. After discharge, although the patient stopped taking the medicines several times, ascites in the follow-up visits were much less than in 2021 and the patient was prone to relapses (Fig. 1).

## Discussion and conclusions

Currently, only two cases of *M. aubagnense* infections have been reported in China and another two in France [3–5]. None of the patients in China had any clinical disease manifestations [3,4]. The first case in France was a 55-year-old man admitted to a hospital with hemoptysis, where the CT scan revealed tuberculosis infection. However, *M. aubagnense* was isolated from his bronchial aspirate. Clarithromycin and amikacin have good clinical effects against *M. aubagnense* infections. The second case was a 91-year-old female who had undergone a revision hip prosthesis surgery for suspected septicemia. She had a fever (39 °C), and the X-ray revealed osteolysis with necrosis and pathological fractures. *M. aubagnense* was isolated from joint fluid collected during surgery. Her condition improved after treatment with amikacin and imipenem [5].

This is the first case report of *M. aubagnense* isolated from ascites. Although the patient was cirrhotic, his liver functions were normal, portal vein pressure was slightly high, and no bacteria or fungi were found in ascites, and the abdominal water volume was very high. Diuretic treatment did not improve his condition, but the abdominal water volume was significantly decreased after anti-NTM treatment (similar to anti-tuberculosis treatment). Thus, the *M. aubagnense* infection was established as the cause of the large number of ascites. This implies that *M. aubagnense* in ascites was the cause of refractory ascites. The patient had recurrent attacks, which were attributed to the underlying cirrhosis. In addition, during anti-NTM treatment, patients often spontaneously stop taking drugs, which they resume after the re-emergence of symptoms. This results in a prolonged treatment cycle, which may decrease the sensitivity of pathogenic bacteria to drugs, resulting in reduced drug efficacy or ineffectiveness. In clinical cases of refractory ascites, the presence of *M. aubagnense* infections or missed diagnoses should be considered. In addition, there is a likelihood of the absence of clinical symptoms when abdominal cavities are only infected with *M. aubagnense*, with clinical symptoms only occurring when the abdominal cavity is complicated with ascites or other celiac diseases. Therefore, *M. aubagnense* infections should be considered in cases of recurring refractory abdominal infection-related diseases.

The low detection rate of *M. aubagnense* is attributed to the low sensitivity and time-consuming nature of the first-generation sequencing technology. Besides, the clinical infection spectrum of *M. aubagnense* overlaps with that of *Mycobacterium mucogenicum*, leading to misdiagnosis in some patients. Moreover, during the initial *M. aubagnense* infection, the clinical manifestations are not apparent as they overlap with those of underlying diseases, which easily results in missed diagnosis. Currently, many non-tuberculous Mycobacterium-causing disease strains are not specifically identified. A rapid differentiation between NTM and *Mycobacterium tuberculosis* complex, including the specific recognition of NTM, is critical for informing clinical treatment and patient management.

## Data Availability

The datasets used and analyzed during the current study are available from the corresponding author upon reasonable request.

## Abbreviations

NTM: Non-tuberculosis mycobacteria
HIV: Human immunodeficiency virus
RGM: Rapidly growing non-tuberculous mycobacteria
*M. aubagnense*: *Mycobacterium aubagnense*
CT: Computed tomography
